# Supplementary material for: Arabidopsis formin 2 regulates cell-to-cell trafficking by capping and stabilizing actin filaments at plasmodesmata
Source: eLife. 2018 Aug 16;7:e36316. doi: 10.7554/eLife.36316 (PMC6126924; doi:10.7554/eLife.36316)
Supplement: Supplementary file 1. [file elife-36316-supp1.docx]

**Supplementary File 1. All primer sequences used in this study.**

| *atfh2-1* LP | CAATTGGTCCAACCAAACAAC |
| --- | --- |
| *atfh2-1* RP | TTCTGTTTCTTAGAGCCGTCG |
| *atfh2-2* LP | AAGTTAGAGCAAGCTCGAGCC |
| *atfh2-2* RP | CTGAGACCTGAAACGACTTGC |
| GK­_LB | ATATTGACCATCATACTCATTGC |
| *atfh1-1* LP | GTCTCCGTCACTGTCGTTAGC |
| *atfh1-1* RP | TTGTTGTTTAACGACTTCGCC |
| *atfh1-3* LP | CGTCTTCATCAGACGAAGAGG |
| *atfh1-3* RP | ATGCTGATACACTGGGGACTG |
| Salk_LB | ATTTTGCCGATTTCGGAAC |
| *AtFH2*_CDSFOR_ | GCGTCGACATGACTACAATACCCTTCTG |
| *AtFH2*_CDSREV_ | TGGGTACCGAACGAGCCATCGTCGTCGT |
| *AtFH1*_CDSFOR_ | ATGCTCTTCTTCTTATTCTTCTTC |
| *AtFH1*_CDSREV_ | TTAAGAAACTAATGAGATTGAG |
| Q1 | ACCACCGTATCAACCACCCG |
| Q2 | TTGCTGGGAGGCTTGGATTG |
| Q3 | GACATCCCTTTCGCCTTTA |
| Q4 | GACCTAGTTTCCGGCATTTA |
| *AtFH2*_PROFOR_ | GCAAGCTTAAAGAGGGGAATGTGTGTGC |
| *AtFH2*_PROREV_ | TGGTCGACTTGCAACGTTTTTCCCCCAA |
| *AtFH2^N437^*_REV_ | TGGGATCCTGGGTCAGAATCATCACCAG |
| *AtFH2^N282^*_REV_ | TGGGATCCAGACGACGAAGCTGGAGGAG |
| *AtFH2^N175^*_REV_ | TGGGATCCACCACCACCACCACCACCGATGATGAAGACGGCGAATA |
| *AtFH2^N282^-ΔTMD*_FOR_ | CGGCCACATCGCTAGGTTCATCCGCCGAACTC |
| *AtFH2^N282^-ΔTMD*_REV_ | GAGTTCGGCGGATGAACCTAGCGATGTGGCCG |
| *AtFH1(N)*_FOR_ | GTCGACATGCTCTTCTTCTTATTCTT |
| *AtFH1(N)*_REV_ | GGATCCACTCGCACAAACCGTCTCTG |
| *AtFH4(N)*_FOR_ | GTCGACATGGCTGCCATGTTGATGCA |
| *AtFH4(N)*_REV_ | GGATCCAGATGATCCTCTAGGAGCCG |
| *AtFH6(N)*_FOR_ | GTCGACATGAAAGCTCTTCAATCCAG |
| *AtFH6(N)*_REV_ | GGATCCACCTGATTTCTCAAGAGAAC |
| *AtFH8(N)*_FOR_ | GTCGACATGGCTGCCATGTTTAATCA |
| *AtFH8(N)*_REV_ | GGATCCTGCTCCTCTAACCGGCGCAG |
| *AtFH9(N)*_FOR_ | GTCGACATGCAGAACTTTTGGTTCGC |
| *AtFH9(N)*_REV_ | GGATCCTACATCATTTTGAACCACAA |
| *AtFH10(N)*_FOR_ | GTCGACATGGACGGACTTTGCTACGT |
| *AtFH10(N)*_REV_ | GGATCCCCCACAACTCTGTGGTAACT |
| *AtFH11(N)*_FOR_ | GTCGACATGGTTTATTTTCGTCAGAT |
| *AtFH11(N)*_REV_ | GGATCCTGGTCTACGCTCAGAAAAAT |
| *AtFH2ΔN_FOR_* | GCGAATTCCGAACTCGCCACCGCCGCCG |
| *AtFH2ΔN*_REV_ | GCGTCGACTTAGAACGAGCCATCGTCGTCT |
| *AtFH2FH1FH2*_FOR_ | GCGAATTCTCTTCTTCTTCTTACTCACA |
| *AtFH2FH1FH2*_REV_ | TGGTCGACTTAGTGGCATGGATCCATACA |
| *AtFH2FH2*_FOR_ | GCGAATTCCATTCCCTTCCTGGTGATGA |
| *AtFH2FH2*_REV_ | TGGTCGACGTGGCATGGATCCATACACCG |
| M1 | GCTTTGTTGAGAGCTCTTAATG |
| M2 | AGCAATGTTGTGTGACTTTCTT |
| M3 | GATGGCGCGGATGGGAAAA |
| M4 | GATATCTACCAGTTTTAAGAGCGTG |
| M5 | GCCCCGGGCCATTCCCTTCCTGGTGATGA |
| M6 | TGGAATTCCTAATGGTGATGGTGATGGTG |
| M7 | GGATCCGAATTCGAGCTCCGTCGACCATTCCCTTCCTGGTGATGATTCTG |
| M8 | CAGTGGTGGTGGTGGTGGTGCTCGAGTGGCATGGATCCATACACCGTTCTC |
| *OsFH8(N)*_FOR_ | TCTAGAATGCCCCCCGCCATCGCGCG |
| *OsFH8(N)*_REV_ | GGATCCTGACGATGAACTATCATCAT |
| *OsFH11(N)*_FOR_ | TCTAGAATGATGAGGCATTGTAGGAGAG |
| *OsFH11(N)*_REV_ | GGATCCCTGCATCGGTGTTGATTCTGAC |
| *OsFH15(N)*_FOR_ | TCTAGAATGCTCGCGCGGTGGCTGCT |
| *OsFH15(N)*_REV_ | GGATCCCGCTCGCTTGTCCTCTGCTCCG |
| *OsFH16(N)*_FOR_ | TCTAGAATGGCTCCGGCTCCGTCTCCGAC |
| *OsFH16(N)*_REV_ | GGATCCCGCGGGTGACGCGACGGCGGC |
| CMV *MP*_REV_ | GGATCCATAAGACCGTTAACCACCTG |
| CMV *MP*-RT_FOR_ | CCCACGGTCGTATTGCTTCC |
| CMV *MP*-RT_REV_ | ACGAAACGCATTGCCCATCT |
| *eIF4A*_FOR_ | CGATGTGCAGCAAGTCTCTC |
| *eIF4A*_REV_ | CTCCCGAACCTTCCACTTCT |
| *HDEL-mCherry*_FOR_ | GCGTCGACATGAAGACTAATC |
| *HDEL-mCherry*_REV_ | GCCTCTAGATTAAAGCTCATCATGAAGC |
