# Supplementary material for: Arabidopsis formin 2 regulates cell-to-cell trafficking by capping and stabilizing actin filaments at plasmodesmata
Source: eLife. 2018 Aug 16;7:e36316. doi: 10.7554/eLife.36316 (PMC6126924; doi:10.7554/eLife.36316)
Supplement: Transparent reporting form [file elife-36316-transrepform.docx]

***eLife’s* transparent reporting form**

We encourage authors to provide detailed information *within their submission* to facilitate the interpretation and replication of experiments. Authors can upload supporting documentation to indicate the use of appropriate reporting guidelines for health-related research (see [EQUATOR Network](http://www.equator-network.org/%20)), life science research (see the [BioSharing Information Resource](https://biosharing.org/)), or the [ARRIVE guidelines](http://www.plosbiology.org/article/info:doi/10.1371/journal.pbio.1000412) for reporting work involving animal research. Where applicable, authors should refer to any relevant reporting standards documents in this form.

If you have any questions, please consult our Journal Policies and/or contact us: [editorial@elifesciences.org](mailto:editorial@elifesciences.org).

**Sample-size estimation**

- You should state whether an appropriate sample size was computed when the study was being designed
- You should state the statistical method of sample size computation and any required assumptions
- If no explicit power analysis was used, you should describe how you decided what sample (replicate) size (number) to use

Please outline where this information can be found within the submission (e.g., sections or figure legends), or explain why this information doesn’t apply to your submission:

GFP and CMV MP-GFP diffusion assays relied on at least three biological replicates. At least 30 cells were analyzed for each replicate.

Fny-CMV inoculation assays relied on at least three biological replicates. At least 30 plants were analyzed for each replicate.

At least 50 data sets were collected for cytoplasmic streaming assays. At least 3 times repeats were performed.

Aniline blue stainning assays relied on at least three biological replicates. At least 100 data sets were analyzed for each replicate.

At least 50 cells were analyzed for actin filaments quantification assays. At least 3 times repeats were performed

Actin nucleation assay, seeded actin elongation assay and actin depolymerization assay relied on at least three biological replicates. At least 3 times repeats were performed for each formin concentration.

Fluorescence light Microscopy of actin filaments assay and TIRFM assay was repeated at least three times and performed with at least 3 times replications for each formin concentration. At least 100 actin filaments were analyzed for each replicate.

**Replicates**

- You should report how often each experiment was performed
- You should include a definition of biological versus technical replication
- The data obtained should be provided and sufficient information should be provided to indicate the number of independent biological and/or technical replicates
- If you encountered any outliers, you should describe how these were handled
- Criteria for exclusion/inclusion of data should be clearly stated
- High-throughput sequence data should be uploaded before submission, with a private link for reviewers provided (these are available from both GEO and ArrayExpress)

Please outline where this information can be found within the submission (e.g., sections or figure legends), or explain why this information doesn’t apply to your submission:

All experiments were performed with at least 3 biological replications.

**Statistical reporting**

- Statistical analysis methods should be described and justified
- Raw data should be presented in figures whenever informative to do so (typically when N per group is less than 10)
- For each experiment, you should identify the statistical tests used, exact values of N, definitions of center, methods of multiple test correction, and dispersion and precision measures (e.g., mean, median, SD, SEM, confidence intervals; and, for the major substantive results, a measure of effect size (e.g., Pearson's r, Cohen's d)
- Report exact p-values wherever possible alongside the summary statistics and 95% confidence intervals. These should be reported for all key questions and not only when the p-value is less than 0.05.

Please outline where this information can be found within the submission (e.g., sections or figure legends), or explain why this information doesn’t apply to your submission:

The mean and standard error were calculated for the Figure 2B,C,D,E,I,J,K; Figure 4B,C,D,I; Figure 5E; Figure 6D; Figure 7G; Figure 1-Figure Supplement 1C; Figure 2-Figure Supplement 1B,C; Figure 2-Figure Supplement 2B; Figure 2-Figure Supplement 3A,D,E; Figure 2-Figure Supplement 4 C,D,F; Figure 4-Figure Supplement 1D,E,F,G; Figure 4-Figure Supplement 2B,C. Unpaired Student's t-test was used for Figure 4I; Figure 5E; Figure 6D; Figure 2-Figure Supplement 2B; Figure 2-Figure Supplement 3D; Figure 2-Figure Supplement 4D; Figure 4-Figure Supplement 1D; Figure 4-Figure Supplement 2B,C. Mann-Whitney U test was used for Figure 2B,C,D,E; Figure 4B,C,D; Figure 7G,J; Figure 2-Figure Supplement 1B,C; Figure 2-Figure Supplement 3E; Figure 4-Figure Supplement 1E; Figure 4-Figure Supplement 2B,C. The information can be found in figure legends and source data.

(For large datasets, or papers with a very large number of statistical tests, you may upload a single table file with tests, Ns, etc., with reference to sections in the manuscript.)

**Group allocation**

- Indicate how samples were allocated into experimental groups (in the case of clinical studies, please specify allocation to treatment method); if randomization was used, please also state if restricted randomization was applied
- Indicate if masking was used during group allocation, data collection and/or data analysis

Please outline where this information can be found within the submission (e.g., sections or figure legends), or explain why this information doesn’t apply to your submission:

**Additional data files (“source data”)**

- We encourage you to upload relevant additional data files, such as numerical data that are represented as a graph in a figure, or as a summary table
- Where provided, these should be in the most useful format, and they can be uploaded as “Source data” files linked to a main figure or table
- Include model definition files including the full list of parameters used
- Include code used for data analysis (e.g., R, MatLab)
- Avoid stating that data files are “available upon request”

Please indicate the figures or tables for which source data files have been provided:

Figure 2 source data 1 is for Figure 2B,

Figure 2 source data 2 is for Figure 2C,

Figure 2 source data 3 is for Figure 2D,

Figure 2 source data 4 is for Figure 2E,

Figure 2 source data 5 is for Figure 2H,

Figure 2 source data 6 is for Figure 2I,

Figure 2 source data 7 is for Figure 2J,

Figure 2 source data 8 is for Figure 2K,

Figure 4 source data 1 is for Figure 4B,

Figure 4 source data 2 is for Figure 4C,

Figure 4 source data 3 is for Figure 4D,

Figure 4 source data 4 is for Figure 4G,

Figure 4 source data 5 is for Figure 4H,

Figure 4 source data 6 is for Figure 4I,

Figure 5 source data 1 is for Figure 5E,

Figure 6 source data 1 is for Figure 6B,

Figure 6 source data 2 is for Figure 6D,

Figure 6 source data 3 is for Figure 6F,

Figure 7 source data 1 is for Figure 7G,

Figure 7 source data 2 is for Figure 7H,

Figure 1-Figure Supplement 1 source data 1 is for Figure 1-Figure Supplement 1C,

Figure 2-Figure Supplement 1 source data 1 is for Figure 2-Figure Supplement 1B,

Figure 2-Figure Supplement 1 source data 2 is for Figure 2-Figure Supplement 1C,

Figure 2-Figure Supplement 2 source data 1 is for Figure 2-Figure Supplement 2B,

Figure 2-Figure Supplement 3 source data 1 is for Figure 2-Figure Supplement 3A,

Figure 2-Figure Supplement 3 source data 2 is for Figure 2-Figure Supplement 3D,

Figure 2-Figure Supplement 3 source data 3 is for Figure 4-Figure Supplement 3E,

Figure 2-Figure Supplement 4 source data 1 is for Figure 2-Figure Supplement 4C,

Figure 2-Figure Supplement 4 source data 2 is for Figure 2-Figure Supplement 4D,

Figure 2-Figure Supplement 4 source data 3 is for Figure 2-Figure Supplement 4E,

Figure 2-Figure Supplement 4 source data 4 is for Figure 4-Figure Supplement 4F,

Figure 4-Figure Supplement 1 source data 1 is for Figure 4-Figure Supplement 1C,

Figure 4-Figure Supplement 1 source data 2 is for Figure 4-Figure Supplement 1D,

Figure 4-Figure Supplement 1 source data 3 is for Figure 4-Figure Supplement 1E,

Figure 4-Figure Supplement 1 source data 4 is for Figure 4-Figure Supplement 1F,

Figure 4-Figure Supplement 1 source data 5 is for Figure 4-Figure Supplement 1G,

Figure 4-Figure Supplement 2 source data 1 is for Figure 4-Figure Supplement 2B,

Figure 4-Figure Supplement 2 source data 2 is for Figure 4-Figure Supplement 2C.
